# Preoperative Risk Stratification of Adnexal Masses: A Narrative Review of Diagnostic Models and the International Ovarian Tumor Analysis (IOTA) Assessment of Different Neoplasias in the Adnexa (ADNEX) Performance

**DOI:** 10.7759/cureus.97027

**Published:** 2025-11-17

**Authors:** Ashika Padival, Bijal M Patel, Chetana D Parekh, Ruchi Arora, Kiran Naik

**Affiliations:** 1 Department of Gynaecologic Oncology, Gujarat Cancer and Research Institute, Ahmedabad, IND; 2 Department of Obstetrics and Gynaecology, Shri Dharmasthala Manjunatheshwara (SDM) College of Health Sciences and Hospital, Dharwad, IND

**Keywords:** accuracy, adnexal masses, diagnosis, iota, ovarian cancer, prediction models, ultrasound

## Abstract

Adnexal masses represent a frequent diagnostic challenge in gynecology, ranging from benign cysts to invasive ovarian malignancies. Accurate preoperative evaluation is crucial for determining appropriate management, surgical planning, and timely referral to oncology centers. Several diagnostic models have been developed to aid this process, including the Risk of Malignancy Index (RMI), Risk of Ovarian Malignancy Algorithm (ROMA), International Ovarian Tumor Analysis (IOTA) Simple Rules, Ovarian-Adnexal Reporting and Data System (O-RADS), and the Assessment of Different Neoplasias in the Adnexa (ADNEX) model. Among these tools, the ADNEX model has gained prominence as a comprehensive framework that distinguishes between benign and malignant tumors and further categorizes malignancies into four types: borderline, early, advanced, and metastatic. This narrative review summarizes the current evidence on diagnostic models for evaluating adnexal masses, highlighting the clinical utility, advantages, and limitations of the IOTA ADNEX model. Available literature consistently indicates that ADNEX demonstrates high diagnostic accuracy and superior risk stratification compared with traditional indices such as RMI and ROMA. The model enhances clinical decision-making by integrating multiple ultrasound and clinical parameters, although challenges persist in operator dependence and in detecting borderline or early-stage malignancies. Continued refinement, context-specific calibration, and integration with biomarkers and advanced imaging techniques are essential for improving diagnostic precision and standardizing global use.

## Introduction and background

Some of the most common diagnostic dilemmas in the field of gynecology include adnexal masses that may be harmless functional cysts or invasive ovarian cancers that demand radical intervention. Globally, adnexal masses are encountered in up to 8%-10% of women during their lifetime, and ovarian cancer remains one of the leading causes of death among gynecologic malignancies, accounting for nearly 3% of all female cancers but the highest mortality rate in this category [1]. The cancer of the ovary is the most fatal of all the gynecological malignancies in the world, and this is because the disease is usually detected at a very late stage due to the vague nature of the symptoms. Thus, proper preoperative distinction of benign and malignant masses is critically important in order to make timely referral to oncology centers and advise proper surgical or conservative care.

Conventional diagnostic techniques are based on the use of ultrasound and serum CA-125; however, separately, these techniques do not have sufficient diagnostic sensitivity. To enhance reliability, various risk prediction models have been established, and they integrate clinical, laboratory, and sonographic data to enable a structured assessment of risks of malignancy. One of the oldest models is the Risk of Malignancy Index (RMI), which combines the menopausal status, CA-125 levels, and the ultrasound characteristics and is only effective in borderline and early-stage cancers [1]. Risk of Ovarian Malignancy Algorithm (ROMA) uses HE4 in addition to CA-125, thus increasing the predictive capacity, but its use is constrained by the availability of the assay type [2].

The International Ovarian Tumor Analysis (IOTA) organization changed the concept of risk stratification by standardizing the terminology of ultrasound and the presentation of diagnostic models of reproducible sonographic characteristics. It has five benign and five malignant ultrasound characteristics that are used in the IOTA Simple Rules (SR) and have been found to have a high diagnostic accuracy, but 10%-20% of cases are indeterminate [3]. To overcome this shortcoming, IOTA proposed logistic regression models and, in 2014, the Assessment of Different Neoplasias in the Adnexa (ADNEX) model. The ADNEX model does estimate not only malignancy but also individual tumor subtypes, such as benign, borderline, stage I, advanced stage, and metastatic disease [4].

The ADNEX model has since been recognized by major professional bodies, including the European Society of Gynaecological Oncology (ESGO), the International Society of Ultrasound in Obstetrics and Gynecology (ISUOG), and the American College of Radiology (ACR), as a valuable tool for preoperative triage and surgical planning [1]. Its integration into diagnostic workflows supports evidence-based referral pathways, ensuring that high-risk patients are directed to oncologic centers while avoiding unnecessary surgical interventions in women with benign lesions.

It has been confirmed in several studies to have a high diagnostic accuracy and be superior to RMI and ROMA [5], and its use is recommended in consensus statements regarding preoperative planning [6]. However, it reduces its accuracy in the assessment of fibromas and borderline tumors, and the diagnostic performance also relies heavily on the experience of the sonographer. Even studies that have the best cut-offs of malignancy differ in the optimal cut-offs of malignancy, further restricting standardization [7]. Therefore, this review aims to synthesize the evidence on diagnostic models of adnexal mass evaluation, with a particular focus on the clinical, methodological, and public health significance of the IOTA ADNEX model.

This was later followed by the introduction of the Ovarian-Adnexal Reporting and Data System (O-RADS), which attempts to standardize the risk categories and management guidelines and is equivalent to Breast Imaging-Reporting and Data System (BI-RADS) in breast imaging [8]. Comparison of O-RADS, ADNEX, and other models has yielded inconsistent results based on population factors, experience of the examiner, and the histology of the tumor [9].

The paper is a narrative review comparing the key diagnostic frameworks: RMI, ROMA, IOTA SR, O-RADS, and ADNEX, and critically appraising published data on the diagnostic performance, strengths, and limitations of these models. The review underlines the clinical relevance of the IOTA ADNEX model and the need to develop it further and conduct more studies.

## Review

In the last 30 years, preoperative evaluation of adnexal masses has changed considerably, and several diagnostic models have been developed that integrate ultrasound characteristics, clinical variables, and biomarkers to distinguish between benign and malignant lesions [10]. Among the most widely used tools are the RMI, the ROMA, the IOTA SR, the O-RADS, and the ADNEX model. Each model builds upon the limitations of its predecessors, progressively improving diagnostic precision and clinical applicability.

RMI, one of the earliest indices, combines menopausal status, ultrasound morphology, and serum CA-125 with a standard cut-off of 200. It remains inexpensive and guideline-supported but shows variable performance among different populations, with limited sensitivity for borderline and early-stage malignancies, restricting its use in fertility-sparing cases [11].

ROMA enhances RMI by incorporating HE4 with CA-125 and adjusting for menopausal status to yield individualized risk scores. Despite improved accuracy, its utility is reduced in premenopausal women and settings with limited access to HE4 assays [3,4,12,13].

The IOTA SR standardize ultrasound interpretation through five benign and five malignant descriptors, achieving high accuracy though leaving 10%-20% of cases indeterminate, which require further expert assessment [5-7,14,15].

O-RADS, developed by the ACR in collaboration with IOTA, provides a categorical risk lexicon (0-5) for structured reporting and management. Its validation is ongoing, and inter-observer variability remains a challenge despite good diagnostic performance [8-11,16,17].

The ADNEX model, introduced in 2014, represents the most comprehensive tool. It combines six ultrasound and three clinical variables, summarized in Table 1, which collectively allow the model to estimate the probability of benign, borderline, stage I, advanced, or metastatic disease [18,19]. It has consistently shown high diagnostic accuracy (area under the curve (AUC) > 0.84) and outperforms RMI and ROMA in multiple validation studies [12-15,20]. Large multicentric studies, including IOTA5, confirm its reproducibility even when applied by less-experienced sonographers, with only minor reductions in accuracy [15-17].

**Table 1:** Input Variables Used in the ADNEX Model and Their Diagnostic Relevance ADNEX: Assessment of Different Neoplasias in the Adnexa

Variable type	ADNEX input variable	Description/diagnostic relevance
Ultrasound predictors (6)	Maximum lesion diameter	Reflects tumor size and complexity.
	Proportion of solid tissue (%)	Distinguishes solid or mixed lesions from cystic ones.
	Number of papillary projections	Indicates epithelial proliferation; higher counts raise malignancy risk.
	Presence of acoustic shadows	Suggests benign composition (e.g., fibroma and dermoid).
	Presence of ascites	Suggests advanced or metastatic disease.
	Number of locules	Indicates cystic complexity and malignancy probability.
Clinical variables (3)	Age (years)	Older age correlates with higher malignancy likelihood.
	Type of center (oncology vs. general)	Adjusts for referral and selection bias.
	Serum CA-125 (U/mL)	Biomarker reflecting tumor activity and disease burden.

As summarized in Table 2, these diagnostic frameworks collectively demonstrate a gradual evolution from basic indices such as RMI and ROMA to sophisticated, multiparameter models like ADNEX, which provide more nuanced risk stratification and subtype differentiation. Table 1 highlights each model’s input variables, strengths, and limitations, underscoring the progressive improvement in diagnostic accuracy over time.

**Table 2:** Comparative Diagnostic Performance of Major Models for Adnexal Masses Compiled and summarized by the authors from previously published studies [1-6,8-15]. RMI: Risk of Malignancy Index; ROMA: Risk of Ovarian Malignancy Algorithm; IOTA: International Ovarian Tumor Analysis; SR: Simple Rules; O-RADS: Ovarian-Adnexal Reporting and Data System; ADNEX: Assessment of Different Neoplasias in the Adnexa; US: ultrasound; AUC: area under the curve

Model	Input variables	Strengths	Limitations	Sensitivity (%)	Specificity (%)	AUC (approx.)	Publication year/region
RMI	CA-125, menopausal status, US morphology	Widely available; guideline-based	Poor in early-stage & borderline tumors	55–71	77–91	0.80–0.82 [1,2]	2022–2023: USA & Europe
ROMA	CA-125, HE4, menopausal status	Incorporates biomarker HE4	Cost; limited HE4 access; population variability	70–75	60–70	~0.75 [3,4]	2023–2024: China & Turkey
IOTA SR	5 benign & 5 malignant US features	Standardized; validated; easy to use	10%–20% indeterminate cases	80–85	80–85	~0.70 [5,6,14,15]	2024–2025: Japan & India
O-RADS	IOTA lexicon + categorical scoring	Standardized reporting and management pathway	Limited validation; examiner variability	85–91	75–83	0.82–0.85 [8-11]	2023–2024: USA, Europe, Asia
ADNEX	6 US + 3 clinical variables	Subclassifies tumors with high accuracy	Requires expertise; less accurate for fibroma/borderline	84–90	83–90	≥0.84 [12-15]	2023–2025: multicenter (Europe, Asia, Latin America)

Overall, ADNEX has become the most comprehensive diagnostic tool with a more detailed stratification compared to the benign/malignant dichotomy. O-RADS has a standard reporting framework, and standardization might be helpful, although IOTA SR may be valuable to general gynecologists. Even though RMI and ROMA are of historical importance, they are gradually being phased away in favor of IOTA-based approaches, as they are more diagnostic. Current opinion tends more toward the utilization of ADNEX and O-RADS as the initial strategy and SR in the low-expert situations.

Synthesis of evidence

Overall Diagnostic Accuracy

International evidence increasingly shows that the IOTA ADNEX model is a valid and flexible diagnostic tool for the preoperative evaluation of adnexal masses. Numerous external validations have demonstrated consistently high diagnostic accuracy, with AUC values typically between 0.82 and 0.90, indicating clear superiority over traditional indices, including the RMI and the ROMA [1-4]. As summarized in Table 2, multicentric validations across various geographic regions reaffirm the model’s reproducibility and diagnostic robustness in diverse populations.

Performance Across Disease Stages

A number of validation studies have found reduced sensitivity of the ADNEX model for borderline and early-stage tumors [21]. These tumors often show overlapping ultrasound characteristics and intermediate biomarker levels compared with benign masses [5,6]. This limitation highlights the importance of careful interpretation in intermediate-risk cases and supports the value of histopathological verification or intraoperative consultation in surgical decision-making [7].

For advanced and metastatic ovarian malignancies, ADNEX demonstrates high specificity and strong predictive performance, with consistent results across large multicentric validations such as the IOTA5 study [8]. This accuracy enhances clinical utility by supporting early referral and improved surgical planning [22].

Influence of Population Characteristics

Population-related differences also influence model performance. Multiple studies have shown that menopausal status affects ADNEX accuracy, with higher specificity in premenopausal women and greater sensitivity in postmenopausal women [9]. These variations reflect physiological differences in tumor morphology and biomarker expression [23]. Therefore, population-specific calibration may further improve model precision across different demographic settings.

Tumor Subtypes and Diagnostic Limitations

According to published data, ADNEX performs effectively in classifying serous and mucinous tumors but shows lower accuracy for fibromas and certain borderline neoplasms [10]. Sonographic similarities between benign solid lesions and low-grade malignancies remain a diagnostic challenge and a potential source of misclassification.

Cut-Off Threshold Optimization

Most external studies suggest that a cut-off value of 20%-30% offers the optimal balance between sensitivity and specificity [11,12]. Lower thresholds increase false positives and are suitable for screening contexts, whereas higher thresholds are preferred for surgical triage and oncology referral [4]. Table 1 summarizes the comparative diagnostic performance of major models, including input variables, strengths, and limitations, illustrating ADNEX’s consistently high AUC across studies.

Comparison With Other Diagnostic Models

In several validation studies, ADNEX has shown superior diagnostic accuracy and classification performance compared with RMI, ROMA, and IOTA SR [13,14]. While O-RADS provides a standardized lexicon and management pathway, its performance may be affected by examiner experience and population variability, leading to inconsistent results when compared with ADNEX [15]. Collectively, these comparisons reinforce ADNEX as a comprehensive and well-validated model for preoperative risk stratification.

Table 3 provides a summary of major external validations of the IOTA ADNEX model in different populations and in different study settings.

**Table 3:** Selected International Validations of the IOTA ADNEX Model Compiled and summarized by the authors from previously published studies [2,6,7,13,16,18,20,24]. IOTA: International Ovarian Tumor Analysis; ADNEX: Assessment of Different Neoplasias in the Adnexa; RMI: Risk of Malignancy Index; ROMA: Risk of Ovarian Malignancy Algorithm; O-RADS: Ovarian-Adnexal Reporting and Data System; AUC: area under the curve

Study/setting	Sample size	Accuracy (%)	Sensitivity (%)	Specificity (%)	AUC	Key notes	Publication year/region
Hiett et al. [7]	400+	86	87	84	0.85	ADNEX is superior to RMI/ROMA	2022: USA (North America)
Vilendecic et al. [2]	350	82	80	85	0.83	ADNEX best among tested models	2023: Serbia (Europe)
Landolfo et al. [6]	>3,000	88	90	86	0.89	Large multicenter validation within IOTA5	2023: Europe (IOTA5 multicenter)
Gaurilcikas et al. [16]	120	70	65	75	0.72	Accuracy reduced in borderline tumors	2020: Lithuania (Europe)
Borges et al. [18]	220	85	86	84	0.86	Prospective external validation; supports two-step strategy	2024: Portugal (Europe)
Shang et al. [20]	80	67	60	75	0.74	Solid tumors often misclassified across models	2023: China (Asia)
Lam Huong et al. [24]	300	83	89	80	0.85	Optimal cut-off ≈ 26 %	2022: Vietnam (Asia)
Almeida et al. [13]	>20 studies	85–90	84–88	82–85	0.87	ADNEX comparable/slightly superior to O-RADS	2025: global (meta-analysis)

Interpretation and clinical implications

Global Diagnostic Reliability

Overall evidence confirms that the IOTA ADNEX model provides high diagnostic accuracy across diverse populations and healthcare settings. Its limitations in fibromas, borderline, and early-stage disease are consistently observed and represent potential areas for refinement. Validation studies conducted across North America, Europe, and Asia show stable performance, highlighting its universal applicability and clinical relevance. In general, the global data support the ADNEX model as a reliable adjunct for preoperative risk assessment of adnexal masses.

Clinical Utility and Patient Triage

Beyond statistical accuracy, the ADNEX model contributes meaningfully to patient triage, surgical decision-making, and the reduction of unnecessary procedures. Combined with complementary tools such as IOTA SR and O-RADS, it enables stratified management of both high- and low-risk adnexal lesions. Proper preoperative risk stratification facilitates timely referral of malignant cases to gynecologic oncologists and the use of conservative or minimally invasive approaches for benign lesions [16,17]. Accurate staging through ADNEX supports surgical planning for cytoreductive procedures, improving survival outcomes [18]. For early-stage or borderline lesions, frozen section or intraoperative consultation remains valuable for fertility-preserving management in younger patients [19].

Operator Expertise and Training

The model’s reliability depends on accurate ultrasound data acquisition and interpretation. Although operator variability remains a concern, studies have shown that sonographers trained in the IOTA lexicon achieve comparable diagnostic accuracy to experienced examiners [20,21]. This supports broader implementation of ADNEX across tertiary and community settings, provided that standardized training and quality assurance are maintained [22].

Integration With O-RADS and Two-Step Strategies

ADNEX can be integrated with O-RADS or used as part of a two-step approach following IOTA SR to improve diagnostic precision. In this strategy, indeterminate cases from SR are subsequently evaluated using ADNEX, reducing false positives and optimizing resource utilization - particularly in low- and middle-income contexts [23,24]. While O-RADS correlates moderately to highly with ADNEX, discrepancies persist in borderline and rare tumor types [25,26].

Performance in Special Populations

ADNEX has shown consistent diagnostic stability in special clinical contexts. This includes women with prior breast cancer, where adnexal lesions may represent metastases [27]. Its reliability has also been demonstrated in Japanese and Thai cohorts, confirming cross-cultural applicability [28,29]. Furthermore, using ADNEX with SR supports safer surgical decision-making in pregnant women by limiting unnecessary interventions where imaging options are restricted [30].

Adjunctive Biomarkers and Imaging Techniques

While ADNEX primarily incorporates CA-125, additional biomarkers such as HE4 can improve early-stage cancer detection [31]. Hybrid approaches combining ADNEX with MRI descriptors have also been proposed to enhance diagnostic precision in indeterminate or complex cases [32].

Healthcare System Implications

Widespread implementation of the ADNEX model can improve healthcare efficiency by decreasing unnecessary laparotomies and optimizing surgical resource allocation. This ensures timely oncologic care for high-risk patients while maintaining safety in conservative management for benign lesions [33,34]. Meta-analyses consistently demonstrate that ADNEX achieves higher AUC values than older indices, reinforcing its inclusion in international clinical guidelines and consensus statements [35,36].

Summary of Clinical Relevance

The ADNEX model combines diagnostic accuracy with real-world applicability, supporting evidence-based referral, personalized surgical planning, and cost-effective healthcare delivery. Its integration with structured ultrasound terminology, operator training, and multimodal strategies ensures that it remains a cornerstone of contemporary preoperative assessment of adnexal masses.

Methodological considerations

This narrative review included peer-reviewed studies published up to 2025, identified through searches in PubMed, Scopus, and Google Scholar using the keywords “ADNEX model,” “O-RADS,” “IOTA,” and “adnexal masses.” Searches were limited to English-language publications. Reference lists of key papers were also screened to capture additional relevant studies.

Multicentric, prospective, and meta-analytic studies were prioritized, as they provide stronger and more generalizable evidence. Retrospective or single-center analyses were included when they offered contextual or population-specific insights but were interpreted with caution. Gray literature, conference abstracts, and preprints were excluded to ensure peer-reviewed reliability.

All statistical data, including sensitivity, specificity, accuracy, and AUC values, were directly extracted from the original studies cited, and confidence intervals were included wherever available. No new statistical analyses were conducted by the authors; all numerical values presented represent secondary data derived from external published sources. The extracted data were reviewed for consistency and accuracy by a biostatistician to ensure methodological rigor and clarity.

Data from the included studies were qualitatively synthesized and presented in comparative tables, focusing on diagnostic accuracy, sensitivity, specificity, and clinical applicability. Studies employing recognized quality-assessment methods, such as QUADAS-2 or equivalent, were given greater interpretive weight. This structured narrative approach ensures methodological transparency and highlights the most robust and contemporary evidence on preoperative risk assessment of adnexal masses.

Limitations and future directions

Although the ADNEX model is well supported by evidence, several factors still limit its universal use. Operator dependence remains an important issue, as accurate measurement of solid areas, papillary projections, and acoustic shadows requires experience. Inter-observer variation persists, and in some cases, expert visual judgment performs better than model-based predictions [37]. Wider adoption of IOTA protocols and focused training programs can help improve consistency across centers [38].

Certain tumor types, such as Brenner tumors, are often misclassified because of overlapping sonographic features [39]. Diagnostic accuracy also decreases in pregnant women and in those with previous surgeries or endometriosis, where MRI scoring or intraoperative frozen section is often required [40]. These special cases highlight the value of combining ADNEX with other imaging or intraoperative techniques.

Choosing the right malignancy-risk cut-off is another challenge. A 10% threshold increases sensitivity but leads to more false positives, while higher values of 20%-30% improve specificity but may miss early cancers. Threshold selection should depend on whether the setting involves screening, triage, or surgical planning [41]. The model’s reliance on CA-125, a non-specific biomarker, also limits accuracy. Adding HE4 or other biomarkers may improve early detection [42].

Future improvements may come from hybrid or artificial intelligence (AI)-assisted versions of ADNEX, such as ADNEX-MR or radiomics-based approaches. These could reduce operator dependence and make assessments more reproducible [39,43].

At present, most validation studies come from Europe and other high-income regions, leaving gaps in data from Asia, South America, and low-resource areas [40,44]. Future research should focus on large multicenter collaborations, inclusion in international guidelines, and assessment of patient outcomes [45]. Broader participation will ensure that ADNEX becomes a truly global and equitable diagnostic tool.

## Conclusions

In gynecologic oncology, preoperative evaluation of adnexal masses plays a vital role in making surgical decisions and referrals. There are various diagnostic models such as RMI, ROMA, IOTA SR, and O-RADS. Among them, the ADNEX model is the most detailed, whereby the tumors are categorized as benign, borderline, early, advanced, or metastatic. Numerous external validations have shown that ADNEX has high diagnostic accuracy and is better stratified than RMI and ROMA. Combined with imaging and biomarker evaluation, the model is a reliable one that has been proven to aid in the clinical decision-making process by differentiating benign and malignant disease. Although it has its strong points, ADNEX is less sensitive to borderline and early-stage malignancies and has a problem with diagnosing fibromas and other solid lesions. To maximize the use of ADNEX, it is important to select malignancy risk cut-offs accurately, and the lower cut-offs should be used with careful management, whereas higher cut-offs should be used with surgical triage. Concomitant with biomarkers, imaging, and clinical expertise, the use of the model is recommended. Further accuracy enhancement can be done through continuous training of the IOTA methodology and population-specific calibration. Enhanced diagnostic performance and expanded clinical applicability of this system might be achieved in the future with integration into AI and other biomarkers.
